# A Modified Run-Off Resistance Score from Cross-Sectional Imaging Discriminates Chronic Critical Limb Ischemia from Intermittent Claudication in Peripheral Arterial Disease

**DOI:** 10.3390/diagnostics12123155

**Published:** 2022-12-14

**Authors:** Jan Paul Frese, Larissa Schawe, Jan Carstens, Karlis Milbergs, Fiona Speichinger, Alexandra Gratl, Andreas Greiner, Ben Raude

**Affiliations:** 1 Department of Vascular Surgery, Charité–Universitätsmedizin Berlin, Hindenburgdamm 30, 12203 Berlin, Germany; 2 Department of Vascular Surgery, Medical University of Innsbruck, Anichstrasse 35, 6020 Innsbruck, Austria

**Keywords:** peripheral arterial disease, run-off resistance, intermittent claudication, chronic critical limb ischemia, angiography, diabetes, atherosclerosis

## Abstract

Atherosclerotic peripheral arterial disease (PAD) leads to intermittent claudication (IC) and may progress into chronic limb-threatening ischemia (CLTI). Scoring systems to determine the atherosclerotic burden of a diseased extremity have been developed. This study aimed to evaluate a modification of the run-off resistance (mROR) score for its usability in cross-sectional imaging. The mROR was determined from preoperative imaging of patients undergoing revascularization for PAD. A total of 20 patients with IC and 20 patients with CLTI were consecutively included. A subgroup analysis for diabetic patients was conducted. The mROR was evaluated for its correlation with disease severity and clinical covariates. Patients with CLTI were older; cardiovascular risk factors, diabetes, and ASA 4 were more frequent. The mROR scores were higher in CLTI than in IC. In diabetic patients, no difference was detected between CLTI and IC. In CLTI, non-diabetic patients had a higher mROR. The mROR score is positively correlated with the severity of PAD and can discriminate CLTI from IC. In diabetic patients with CLTI, the mROR is lower than in non-diabetic patients. The mROR score can be determined from cross-sectional imaging angiographies. It may be useful for clinicians helping with vascular case planning, as well as for scientific purposes.

## 1. Introduction

Atherosclerotic cardiovascular diseases are the leading cause of death worldwide, with increasing incidences over the past few decades. One such manifestation is the peripheral arterial disease (PAD), affecting more than 200 million people worldwide [1]. Men are affected more frequently than women, with an increased incidence after the age of 65. Besides smoking, diabetes mellitus (DM) is one of the main risk factors for PAD and the progression of the disease [2,3].

Atherosclerotic lesions within the arterial tree from the aorta to the lower extremities are the hallmarks of PAD [4]. Initially asymptomatic, the chronic progression of atherosclerotic stenosis causes exercise-dependent pain in the state of intermittent claudication (IC) and may lead to the most severe state of chronic critical limb-threatening ischemia (CLTI) [5]. Frequently, multilocular atherosclerotic lesions with different degrees of severity are present. To improve the quality of life in IC and to prevent limb amputation in CLTI, treatment strategies include endovascular, open surgical, or combined (“hybrid”) techniques [6]. Treatment strategies are based on preoperative vascular imaging and consider the severity of symptoms, as well as the anatomical configuration of the disease in individual patients. Existing classification systems in PAD are based on symptoms or anatomical patterns [7] and aim to facilitate treatment decisions, such as in the latest Trans-Atlantic Inter-Society Consensus Document on Management of Peripheral Arterial Disease (TASC II) [8,9], or are targeting specific stages of the disease, such as the Bollinger system [10] used in the Bypass versus Angioplasty in Severe Ischemia of the Leg (BASIL) study [11] and the Global Limb Anatomic Staging System (GLASS) [12]. A scoring tool to describe the atherosclerotic burden usually includes the degree of stenosis, the length of the lesion, and the arterial level(s) that are affected. It generates numeric values, which correlate with the severity of the disease and which are helpful for surgical decision-making. Furthermore, they are used to objectively describe and compare PAD patients in studies. In this study, we used a modification of the run-off resistance (ROR) score proposed by Peterkin et al. 1988 [13]. The aim of this study was to evaluate the run-off resistance score for its usability in cross-sectional imaging to discriminate IC from CLTI cases in peripheral arterial disease.

## 2. Materials and Methods

### 2.1. Study Population

In this single-center study from a tertiary vascular unit, 40 consecutive patients with peripheral arterial disease (PAD) were included. Two subgroups of PAD, each including 20 patients, were defined: patients with intermittent claudication (IC) and an indication for open- or endovascular revascularization (Rutherford stage 3), and chronic limb-threatening ischemia (CLTI). CLTI comprised Rutherford stages 4 to 6, defined by resting pain, necrotic non-venous ulcers, or gangrene. Preoperative case planning was conducted with computed tomography angiographies (CT-A) or magnetic resonance imaging angiographies (MR-A), respectively. Exclusion criteria were: age < 18 years, pregnancy, transplantation, emergency interventions, major amputations, and inability to give informed consent. Medical history and anthropometric parameters including sex, age, body mass index (BMI), diabetic status, and cardiovascular risk factors were recorded. The physical examination included inspection for skin lesions at the lower extremities, pulse status, and ankle–brachial index (ABI). If applicable, the maximum walking distance was determined by a treadmill test at a continuous speed of 3 km per hour and an inclination of 10%.

### 2.2. Ethics

The institutions’ medical ethical committee approved the protocol and the design of this study under proposal number EA4/139/16. All participants gave their written consent. All procedures were in accordance with the 1964 Helsinki Declaration and its later amendments.

### 2.3. Modified Run-Off Resistance Score (mROR)

**Figure 1:**
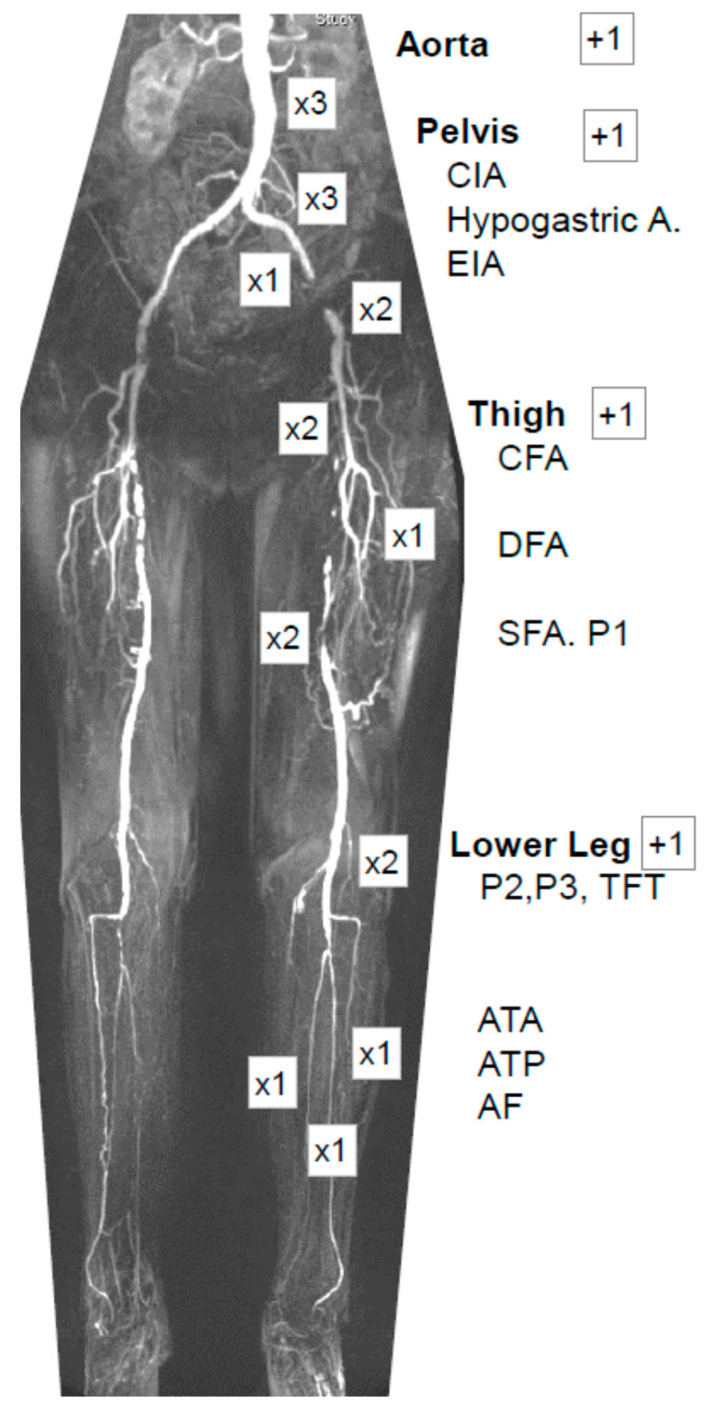
Multiplanar reconstruction of an MR-A of a patient with severe arterial disease. The main levels constituting the modified runoff resistance score (mROR) are the infrarenal aorta, pelvis, thigh, and lower leg. Each vessel is graded by the degree of stenosis (1 for 20–49% stenosis, 2 for 50–99% stenosis, 2.5 for occlusion of less than half the length, and 3 for occlusion of more than half the length of the vessel) and multiplied with its weighting factor depicted. For each main level, 1 is added as the physiologic runoff resistance. CIA: common iliac artery; EIA: external iliac; CFA: common femoral; DFA: deep femoral; SFA: superficial femoral arteries; P1, P2, P3: popliteal artery, segments 1–3; TFT: tibiofibular trunk; ATA: anterior tibial; ATP: posterior tibial; AF: peroneal artery.

The original run-off resistance Score (ROR) proposed by Peterkin et al. [13] originated from the pre-endovascular era and was based on conventional angiographies. The score showed the degree and quality of arteries distally of a proposed femoropopliteal bypass site in order to predict the prognosis of the planned surgery. Based on the Reporting Standards for the Society for Vascular Surgery and the International Society for Cardiovascular Surgery angiographic scoring system [14], each vascular segment was graded by the degree of stenosis or occlusion and the length of atherosclerotic lesions, combined with an arbitrary weighting factor for each arterial level. The multipliers for the degree of stenosis were 1 for 20–49% stenosis, 2 for 50–99% stenosis, 2.5 for occlusion of less than half the length, and 3 for occlusion of more than half the length of the vessel segment. The ROR sum score was calculated by the addition of the multipliers for each vessel, multiplied with the weighting factor for each vascular segment shown in Figure 1 and adding 1 to each segment as the intrinsic run-off resistance of a non-diseased segment. ROR was later modified for use with MR-A [15], evaluated for study purposes [16], and showed a good predictive value for clinical outcome parameters, such as the ankle–brachial pressure index (ABI) [17]. The original score was developed for below-the-knee bypass surgery and lacked a weighting factor for the popliteal segment, which we added to provide more descriptive accuracy. Thus, the modified score (mROR) provided an accurate linear value for the description of the atherosclerotic burden of the arterial tree in patients with PAD. Two vascular surgeons who were blinded for the disease stage and intervention evaluated the CT-A or MR-A independently and calculated the mROR score for the diseased extremities.

### 2.4. Statistics

Statistical analysis was carried out using SPSS 27 (IBM, Armonk, NY, USA). Continuous data were given as the mean ± standard deviation in case of Gaussian distribution or as the mean with 95% confidence interval (in brackets) in case of a non-Gaussian distribution and are presented as box-plots, including median and lower/upper quartiles; whiskers denote 5% and 95% percentiles. *p*-values < 0.05 were considered significant.

Average age and sex frequency were calculated. To compare column means, a non-parametric (Mann–Whitney U) test was used. To exclude a bias of age and body mass index (BMI) on the assessed parameters, a linear regression analysis was carried out. As a primary aim, the mROR scores in the IC and CLTI groups were compared. A post-hoc subgroup analysis of diabetic status was performed.

## 3. Results

### 3.1. Patient Characteristics

Of the 40 patients included, 28 (70%) were male. The 20 CLTI patients were older than those with IC (CLTI: 70.7 years, IC 63.0 years, *p* = 0.010). CLTI patients had more cardiovascular risk factors (CLTI: 3.0, IC: 2.0, *p* = 0.047). ASA 4 and DM were more frequent in CLTI (ASA 4: 30% and 0%, *p* = 0.005; DM: 50% and 15%, *p* = 0.005). The ABI was not different between CLTI and IC. When diabetic patients were excluded, ABI was 0.62 ± 0.18 in IC and 0.42 ± 0.28 in CLTI; however, this difference was not significant (*p* = 0.183). Treatment strategies in the CLTI group included open surgery in twelve patients (60%), endovascular intervention in two patients (10%), and hybrid operating techniques in six patients (30%). In the IC group, treatment strategies included eight (40%) open, four (20%) endovascular, and eight (40%) hybrid therapies; however, this difference was not statistically significant (*p* = 0.416). Epidemiologic details are given in Table 1.

### 3.2. mROR Sum Scores and Subgroup Analysis

The sum scores of mROR in the main arterial levels for the study population and the subgroups of CLTI, IC, diabetics, and non-diabetics are given in Table 2. In the whole study cohort, and even more markedly in the non-diabetic subgroup, CLTI had higher mROR scores than IC patients in the thigh (*p* = 0.0029) and lower leg (*p* = 0.001), resulting in a higher total score (*p* = 0.004) (correlation coefficient for CLTI: r = 0.462, *p* = 0.001). In diabetic patients, no difference was found between CLTI and IC. In IC, no difference was found between diabetic and non-diabetic patients. In CLTI, however, non-diabetic patients showed a higher mROR score in the pelvis (*p* = 0.004) and lower leg (*p* = 0.033), resulting in a higher total score (*p* = 0.001).

**Figure 2:**
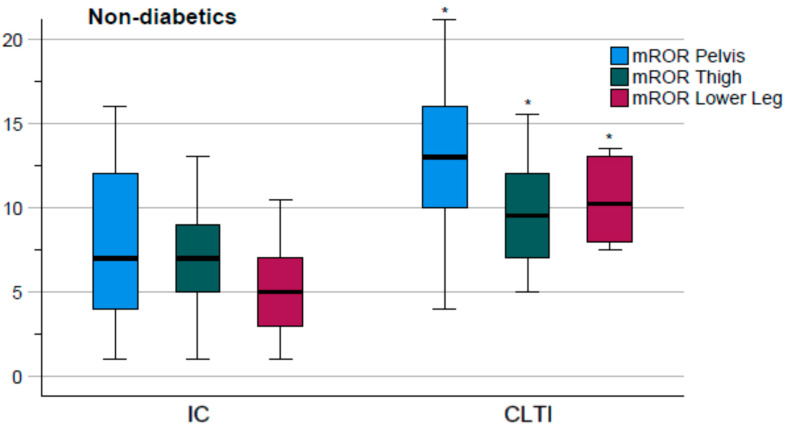
Subgroup analysis of the modified runoff resistance (mROR) scores of the three main arterial levels of intermittent claudication (IC) and chronic limb-threatening ischemia (CLTI) in non-diabetic patients. An asterisk * marks a significant difference.

As no difference between IC and CLTI was present in diabetic patients, those patients were excluded from the detailed analysis shown in Table 3. Among patients without DM, CLTI patients had higher mROR in the main segments values than IC patients (pelvis *p* = 0.029, thigh *p* = 0.038, and lower leg *p* = 0.001), as depicted in Figure 2.

### 3.3. Univariate Regression Analysis

Linear regression analysis for the parameters given in Table 1 was carried out. The results of univariate regression are shown in Table 4. In the whole study cohort, CLTI was associated with a higher mROR (B = 8.28, *p* = 0.003). In the CLTI group, the body mass index and diabetes were inversely correlated to mROR (BMI: B = −0.77, *p* = 0.023; diabetes: B = −12.2, *p* = 0.001).

## 4. Discussion

Several studies have investigated the correlation between angiographic imaging and clinical parameters in PAD. Scoring systems have been developed to derive therapeutic decisions or for scientific purposes [10,12,18]. The original runoff resistance score (ROR) [13,14] was determined from conventional angiographies and was later modified and evaluated for MR-A [15,16], demonstrating a good correlation with clinical outcome parameters [19]. ROR was better correlated with ABI than other scoring systems, but ABI is of limited significance in diabetic patients [17,20]. The aim of this study was to evaluate the modified score for its practicability in cross-sectional imaging and to determine its correlation with disease severity and clinical parameters. The modified score we used was generated from the preoperative CT-As and MR-As of PAD patients undergoing open or endovascular revascularization. This score aimed to provide a linear value for the impairment of peripheral perfusion, weighted by the location and the severity of atherosclerotic lesions in the arterial tree of a diseased extremity. This could be useful for case planning, but also the monitoring, of disease progression during follow-up.

CLTI patients in our study were older than IC patients and had more cardiovascular risk factors and a higher ASA category, which is in line with the natural history of PAD [4]. A significant correlation between mROR and disease severity was found: the sum scores of mROR in the main arterial levels were higher in CLTI than in IC. The Bollinger angiographic scoring system was evaluated for IC [21] and was modified to include and evaluate the distal arteries in diabetic patients later [22]. A comparison of disease severity is missing. The more recent Global Limb Anatomic Staging System (GLASS) [12] was proposed for CLTI. The scoring system is similar to the score proposed by Morris [18], but in this score, the hypogastric artery is missing, which could serve as a collateral network, especially in aortoiliac PAD. With this study, we were able to conduct a subgroup analysis of two relevant clinical stages of PAD in diabetic and non-diabetic patients. Patients with CTLI had higher scores in all levels of the arterial tree, not just in the distal arteries. It can be argued that the disease progression from IC to CLTI is the sum of a multilevel process. In IC patients, there was no difference between diabetic and non-diabetic patients; thus, the symptomatology of IC, and as a consequence, the indication for invasive treatment, did not differ. In contrast, and most interestingly, CLTI patients with diabetes did not have higher but lower mROR scores in the pelvic and lower leg regions than non-diabetic CLTI patients. A patient with PAD in combination with diabetes is more likely to develop chronic critical ischemia by concomitant diabetic angiopathy [23]. Our mROR results demonstrate this by suggesting that a combination of atherosclerotic macroangiopathy with diabetic microangiopathy rather than a more progressive atherosclerotic macroangiopathy is the trigger for progression from IC to CLTI in diabetic patients. The indication for treatment and the therapeutic strategy of PAD in patients with DM is not different from the treatment of non-diabetic patients. As one limitation of our study, the prevalence of diabetes was not balanced in the IC and CLTI cohorts. Diabetic status and mROR were evaluated as a post-hoc subgroup analysis in this study. In the CLTI group, the prevalence of diabetes was 50%, which is higher than in the Framingham study PAD subgroup of 20% [24].

## 5. Conclusions

In summary, the mROR score is positively correlated with the severity of PAD and can discriminate CLTI from IC. In diabetic patients with CLTI, the mROR is lower than in non-diabetic patients. The mROR score can be determined from cross-sectional imaging angiographies. It may be useful for clinicians helping with vascular case planning, as well as for scientific purposes.

## Figures and Tables

**Table 1:** Overview of anthropometric data of the study cohort of patients with peripheral arterial disease (PAD) in the stage of intermittent claudication (IC) or chronic limb-threatening ischemia (CLTI). Categorical variables are expressed as numbers and percentages in brackets. Continuous variables are expressed as the median with the interquartile range (IQR) in brackets. *p*-values were generated with non-parametric Mann–Whitney U tests. An asterisk * marks a *p*-value < 0.05. ASA: American Society of Anesthesiologists’ comorbidity scale.

	IC (*n* = 20)		CLTI (*n* = 20)		*p*-Value
		(%) (IQR)		(%) (IQR)	
male gender	15	(75)	13	(65)	0.490
age [years]	63.0	(59.7–72.3)	70.7	(66.1–79.5)	0.010 *
PAD Rutherford stage	2.0	(2.0–3.0)	5.0	(5.0–5.0)	0.000 *
body mass index (BMI)	27.2	(20.9–28.9)	26.1	(24.1–31.9)	0.365
overweight (BMI > 25)	12	(60)	12	(60)	1.000
ASA 2	9	(45)	2	(10)	
ASA 3	11	(55)	12	(60)	
ASA 4	0	(0)	6	(30)	0.005 *
Diabetes	3	(15)	10	(50)	0.018 *
Hypertension	15	(75)	18	(90)	0.212
active smoker	13	(65)	10	(50)	0.337
coronary heart disease	5	(25)	11	(55)	0.053
dyslipoproteinemia	13	(65)	12	(60)	0.744
number of cardiovascular risk factors	2.0	(1.0–2.5)	3.0	(2.0–4.5)	0.047 *
kidney disease	4	(20)	11	(55)	0.022 *
permanent dialysis	0	(0)	2	(10)	0.147
ankle-brachial index (ABI) before revascularization	0.6	(0.5–0.8)	0.8	(0.5–1.0)	0.571
technique of revascularization					
− Open	8	(40)	12	(60)	
− Endovascular	4	(20)	2	(10)	
− Hybrid	8	(40)	6	(30)	0.416

**Table 2:** Sum scores and subgroup analyses of the main arterial levels (pelvis including the infrarenal aorta, thigh, and lower leg) of the modified runoff resistance (mROR) anatomical scoring system acquired by computed tomography angiography; comparing groups of patients with intermittent claudication (IC) or chronic limb-threatening ischemia (CLTI) and diabetic or non-diabetic patients. ROR values are given as mean ± standard deviation. An asterisk * marks a *p*-value < 0.05.

	*n*	mROR Pelvis		mROR Thigh		mROR Lower Leg		mROR Sum	
**study cohort**	40	9.62	±5.15	8.09	±3.28	7.53	±3.61	27.59	±9.06
− IC	20	8.90	±4.88	6.88	±3.44	5.78	±3.82	23.45	±7.17
− CLTI	20	10.35	±5.45	9.30	±2.68	9.28	±2.38	31.73	±9.01
− *p*-value			0.432		0.029 *		0.001 *		0.004 *
**IC**									
− diabetics	3	8.00	±6.56	8.67	±0.58	7.00	±5.57	25.67	±12.42
− non-diabetics	17	9.06	±4.76	6.56	±3.65	5.56	±3.62	23.06	±6.38
− *p*-value			0.750		0.309		0.670		0.874
**CLTI**									
− diabetics	10	6.90	±3.41	8.80	±2.25	8.05	±1.64	25.65	±4.96
− non-diabetics	10	13.80	±4.95	9.80	±3.09	10.50	±2.44	37.80	±8.05
− *p*-value			0.004 *		0.493		0.033 *		0.001 *
**diabetics**									
− IC	3	8.00	±6.56	8.67	±0.58	7.00	±5.57	25.67	±12.42
− CLTI	10	6.90	±3.41	8.80	±2.25	8.05	±1.64	25.65	±4.96
− *p*-value			0.798		0.729		0.550		0.612
**non-diabetics**									
− IC	17	9.06	±4.76	6.56	±3.65	5.56	±3.62	23.06	±6.38
− CLTI	10	13.80	±4.95	9.80	±3.09	10.50	±2.44	37.80	±8.05
− *p*-value			0.029 *		0.038 *		0.001 *		<0.001 *

**Table 3:** Modified run-off resistance (mROR) scores per arterial region in three anatomical levels (pelvis, thigh, and lower leg) in non-diabetic patients with intermittent claudication (IC) or chronic limb-threatening ischemia (CLTI). The mROR values are given as the mean ± standard deviation. An asterisk * marks a *p*-value < 0.05.

	IC		CLTI		*p*-Value
	*n* = 17		*n* = 10		
Aorta	1.88	±1.41	3.70	±1.70	0.009 *
Common iliac artery	3.00	±2.81	4.05	±3.00	0.358
Hypogastric artery	0.82	±0.95	1.65	±0.85	0.028 *
External iliac artery	2.35	±1.62	3.40	±1.35	0.086
**Sum mROR pelvis**	8.06	±4.76	12.80	±4.95	0.029 *
Common femoral artery	2.24	±1.71	3.00	±1.70	0.324
Deep femoral artery	0.50	±0.79	1.10	±1.07	0.133
Superficial femoral artery and supragenual popliteal artery (P1)	2.82	±2.13	4.70	±1.64	0.028 *
**Sum mROR thigh**	6.56	±3.65	9.80	±3.09	0.038 *
Infragenual popliteal artery (P2–P3) and tibiofibular trunk	0.94	±1.09	2.00	±0.78	0.014 *
Anterior tibial artery	1.06	±0.97	1.90	±0.70	0.025 *
Posterior tibial artery	0.79	±0.95	2.05	±0.93	0.004 *
Peroneal artery	0.82	±1.17	1.55	±1.01	0.054
**Sum mROR lower leg**	5.56	±3.62	10.50	±2.44	0.001 *
**Sum mROR**	23.06	±6.38	37.80	±8.05	<0.001 *

**Table 4:** Significant predictor variables from univariate linear regression analyses for the total sum score of the modified runoff resistance (mROR) score for the study cohort of 40 patients and the subgroup of 20 patients with chronic limb-threatening ischemia (CLTI). All epidemiologic parameters from Table 1 were tested for their predictive values to mROR. In the subgroup of intermittent claudication (IC), no significant predictor variable was found. The coefficient for the continuous variables Rutherford stage and body mass index refer to a change of one unit. A negative value signifies a reverse correlation. An asterisk * marks a *p*-value < 0.05. B: regression coefficient B; CI: confidence interval.

Study Cohort		B	95% CI	*p*-Value
	CLTI over IC	8.28	(3.06–13.49)	0.003 *
	Rutherford stage	2.20	(0.24–4.16)	0.029 *
**CLTI subgroup**				
	body mass index (BMI)	−0.77	(−1.42–−0.12)	0.023 *
	Diabetes	−12.2	(−18.43–−5.87)	0.001 *

## Data Availability

Data are contained within the article or supplementary material. The data presented in this study are available upon request from the corresponding author.
